# Endoscopic Ultrasound With Fine Needle Biopsy Confirming a Diagnosis of Immune Checkpoint Inhibitor-Related Type 3 Autoimmune Pancreatitis

**DOI:** 10.1155/crgm/5519015

**Published:** 2025-10-31

**Authors:** Dana Ley, Kusum Sharma, Saqib Walayat, Mark R. Albertini, Rashmi M. Agni, Deepak V. Gopal

**Affiliations:** ^1^Division of Gastroenterology and Hepatology, Department of Medicine, University of Wisconsin School of Medicine and Public Health, Madison, Wisconsin, USA; ^2^Department of Pathology and Laboratory Medicine, University of Wisconsin School of Medicine and Public Health, Madison, Wisconsin, USA; ^3^Division of Hematology, Oncology, and Palliative Care, Department of Medicine, University of Wisconsin School of Medicine and Public Health, Madison, Wisconsin, USA; ^4^Department of Dermatology, University of Wisconsin School of Medicine and Public Health, Madison, Wisconsin, USA

**Keywords:** endoscopic ultrasound, fine needle biopsy, immune checkpoint inhibitor-related pancreatitis, metastatic melanoma, type 3 autoimmune pancreatitis

## Abstract

**Introduction:**

Immune checkpoint inhibitor-related pancreatitis, also known as type 3 autoimmune pancreatitis (AIP), is uncommon and has a widely ranging clinical presentation. We present the biopsy findings of a case consistent with type 3 AIP—an entity recently described in the literature, the pathologic findings of which have not been well characterized.

**Case Report:**

A 71-year-old male with metastatic mucosal melanoma of the urethra was treated with immune checkpoint inhibitor (ICI) therapy (nivolumab/relatlimab) and developed vague epigastric discomfort. He was found to have an elevated lipase, which increased to > 20x the upper limit of normal. Subsequent imaging showed new infiltrative masses in the pancreatic head and distal body/tail. Endoscopic ultrasound with fine needle biopsy (FNB) was performed. This showed T-lymphocyte predominant infiltrates, in the acini and septal areas, with concomitant acinar, duct, and venular damage, including both CD4 and CD8 lymphocytes, which were considered consistent with type 3 AIP. He was treated successfully with prednisone.

**Discussion:**

On biopsy, there was no evidence of malignancy or features of type 1 or type 2 AIP. Histologic findings included moderate infiltration and damage to the pancreatic parenchyma, ductal, and vascular structures by CD4 and CD8 lymphocytes, pointing to immune-mediated pancreatic injury, and supportive of ICI-mediated injury to the pancreas of this patient. The clinical presentation of type 3 AIP ranges from asymptomatic lipase elevation to asymptomatic pancreatitis to acute symptomatic pancreatitis. There may be no clear temporal relationship to treatment initiation. Type 3 AIP typically presents along with other immune-related adverse events. Endoscopic ultrasound with FNB contributed to diagnostic certainty in this case and changed our patient's management, allowing for appropriate treatment of his immune-related adverse event.

## 1. Introduction

Immune checkpoint inhibitors (ICIs) are immune-modulating antibodies used to enhance the immune system. These include monoclonal antibodies that block cytotoxic T lymphocyte-associated protein 4 (CTLA-4), programmed cell death receptor 1 (PD-1), and programmed cell death ligand 1 (PD-L1) [1, 2]. ICIs are used to treat multiple advanced malignancies including metastatic melanoma and have changed the standard of care and significantly improved outcomes for many patients [3]. Despite being highly effective, ICIs may lead to immune-related adverse events (irAEs) that may affect any organ system and vary in severity.

ICI-related pancreatitis, also called type 3 autoimmune pancreatitis (AIP), is uncommon and may have a variable presentation. The incidence of type 3 AIP of all severities is estimated to range from 0.6% to 4%, and it nearly always occurs along with other irAEs [4, 5]. It most often appears about 3-4 months after ICI exposure [6]. ICI-related pancreatitis exists on a clinical spectrum and may present with asymptomatic lipase elevations, incidental imaging findings of pancreatitis, or symptomatic pancreatitis. It is most common (in about two-thirds of cases) for patients to present clinically asymptomatic [6].

Management for patients with ICI-related pancreatitis is primarily supportive, including intravenous hydration, pain medications, and holding the ICI [7]. Corticosteroid therapy may be given based on the severity of the pancreatitis. However, there is some evidence that pancreatic injury may improve on its own without corticosteroid therapy [8]. In some cases, the ICI therapy may be resumed. Some retrospective data suggest that patients with ICI-related pancreatitis may be rechallenged with ICI therapy and have a low likelihood of relapse [8]. However, in severe cases of ICI-related pancreatitis, the ICI may be discontinued permanently [9, 10].

To date, most cases have been diagnosed based on clinical features and imaging findings and have usually been treated without obtaining a biopsy. We present a case consistent with ICI-related pancreatitis, with a detailed description of pathologic findings, compared with findings of ICI-related injury in other organs such as the liver and to other types of AIP.

## 2. Case Report

A 71-year-old male with metastatic mucosal melanoma of the urethra was treated with ICI therapy (Opdualag, a fixed combination of nivolumab 480 mg and relatlimab 160 mg, given intravenously once every 4 weeks), complicated by adrenal insufficiency and microscopic colitis. After cycle four of ICI therapy, he developed vague epigastric discomfort and was found to have an elevated lipase (< 5x the upper limit of normal [ULN]). His serum cortisol and thyroid hormone labs were being surveilled, and after cycle four, his morning cortisol level was found to be low at 1.4 mcg/dL (reference range: 3.7–19.4 mcg/dL). Follow-up testing a few days later demonstrated an undetectable cortisol level and low adrenocorticotropic hormone (ACTH) level of 4 pg/mL (reference range: 7.2–63.3 pg/mL). He had had no recent significant exogenous corticosteroid exposure. Due to abnormal adrenal labs and symptoms which included headache and nausea, he went to the emergency department and had a brain MRI. This showed normal appearance of the pituitary gland. He was referred to endocrinology and was deemed to have secondary adrenal insufficiency. He was started on hydrocortisone (initially 20 mg in the morning, 10 mg in the afternoon, subsequently weaning down to 10 mg in the morning, and 5 mg in the afternoon).

**Figure 1:**
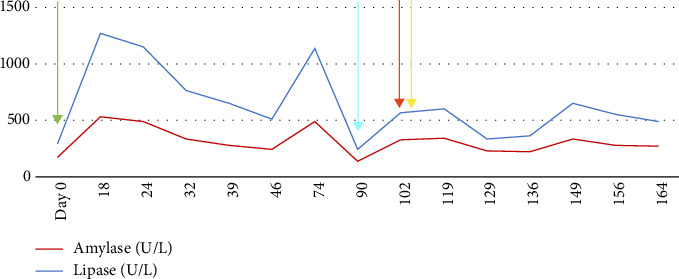
Patient's amylase and lipase trends in units/L. Clinically significant time points are indicated by the arrows. The green arrow at Day 0 indicates when cycle five of ICI therapy was administered. The ICI was not given again after this time point. The cyan arrow is located at approximately Day 91 and indicates when the initial abnormal-appearing CT scan of the abdomen and pelvis was obtained. The red arrow is located at approximately Day 101, which is when the EUS with FNB was performed. The yellow arrow is located at approximately Day 103, which is when the patient was hospitalized with acute pancreatitis and started on prednisone.

After cycle five, the lipase further increased to > 20x ULN (Figure 1). Around this timeframe, he also developed diarrhea, with up to 8 loose stools daily. He underwent a colonoscopy that showed a grossly normal-appearing colon and terminal ileum. Random biopsies were taken throughout the entire colon, and pathology was consistent with microscopic (lymphocytic) colitis. Due to ICI-related microscopic colitis and concern for ICI-related pancreatitis, his ICI was held. For the microscopic colitis, he was started on budesonide in addition to the hydrocortisone for secondary adrenal insufficiency.

**Figure 2:**
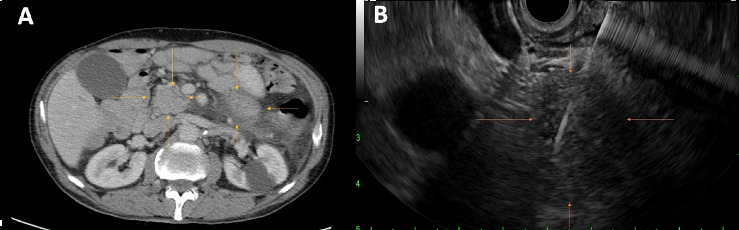
(A) Axial computed tomography (CT) of the abdomen and pelvis with contrast. Arrows indicate infiltrative masses in the pancreatic head and distal body/tail. (B) Endoscopic ultrasound (EUS) with fine needle biopsy (FNB) image. Arrows indicate irregular oval mass in pancreatic head, 30 mm × 18 mm in maximal cross-sectional diameter with irregular outer margins. 22-gauge core needle is seen entering the mass.

One month after ICI was held, computed tomography of the abdomen and pelvis (CT A/P) with contrast showed a prominent pancreatic duct without clear mass. Positron emission tomography (PET) scan was obtained, showing improvement in prior metastatic inguinal lymph node uptake. Three months later, the amylase and lipase remained elevated and began to uptrend (Figure 1). Repeat CT A/P with contrast was performed and showed new infiltrative masses in the pancreatic head and distal body/tail (Figure 2(a)). Immunoglobulin G4 (IgG4) level was checked and was normal.

**Figure 3:**
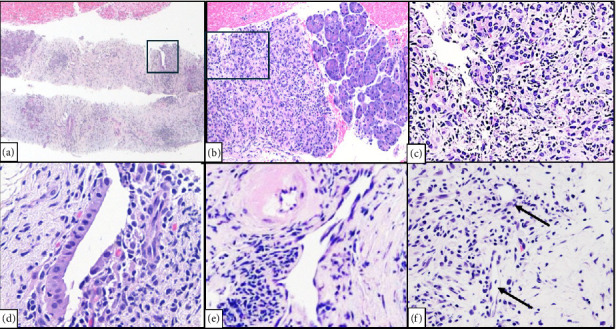
Photomicrographs of pancreatic needle core biopsy H&E stain. (a) Extensive parenchymal inflammation with acinar dropout and patchy stromal/septal inflammation, accentuated around intralobular ducts (within rectangle), and vessels (40x magnification). (b) Patchy lobular mixed inflammation with acinar dropout in left half of image contrasted with intact noninflamed acini on the right (100x magnification). (c) High power view of area within rectangle in (b) showing lymphocyte-rich inflammation admixed with neutrophils, infiltrating acini, and stroma. (d) Septal periductal inflammation focused in and around the interlobular pancreatic duct shown within the area of the rectangle in (a). Note the lymphocytes surrounding and infiltrating within duct epithelium and an area of duct epithelial disruption due to lymphocyte-mediated injury. (e) Septal perivenular lymphocyte-rich inflammation around a venule with swollen endothelial cells and an adjacent uninvolved artery with hyalinized wall. (f) Septal perivenular inflammation with aggregates of small lymphocytes observed around small vessels (involved venules, indicated by black arrows, are cuffed and infiltrated by lymphocytes, with lifting up of endothelial cell nuclei) (images (c–f) at 400x magnification).

**Figure 4:**
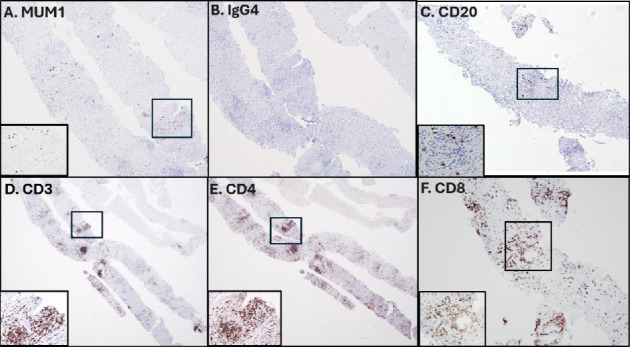
Photomicrographs depicting the immunohistochemical characterization of the inflammatory infiltrate within the pancreatic parenchyma focusing on septal periductal infiltrates (background images of (A–C and F) at 100x magnification; (D-E) background images at 40x magnification; insets in (A and C–F) at 400x magnification).

Endoscopic ultrasound (EUS) showed two hypoechoic irregular-appearing masses in the pancreatic head and distal body with upstream pancreatic ductal dilation (Figure 2(b)). Fine needle biopsy (FNB) of the masses (Figures 3(a), 3(b), 3(c), 3(d), 3(e), 3(f), 4(a), 4(b), 4(c), 4(d), 4(e), and 4(f)) revealed pancreatic parenchymal cores in which the acini were infiltrated by a moderate lymphocyte-predominant mixed inflammatory infiltrate, with acinar damage and dropout in a patchy distribution. Neutrophils were present in small numbers, in addition to rare eosinophils, plasma cells, and histiocytes. Within the interlobular septa, there were prominent lymphocyte-rich mixed inflammatory infiltrates, with conspicuous cuffing around ducts and vascular structures. In addition, there was intraductal lymphocytic infiltration, associated with duct epithelial damage and nuclear dropout, as well as venous endothelial inflammation, with endothelial cell swelling and lymphocytes in the subendothelial space. By immunohistochemistry (IHC), the lymphocytes stained for CD3 (T-lymphocyte marker) with only rare CD20 cells (B-cell marker). The T-lymphocytes coexpressed either CD8 (cytotoxic T-cell marker), or CD4 (helper T-cell marker), both present in fair to moderate numbers. MUM1, a plasma cell marker, depicted rare plasma cells, of which none were reactive for IgG4. These histologic and immunohistochemical findings were deemed consistent with the diagnosis of ICI-related pancreatitis.

**Figure 5:**
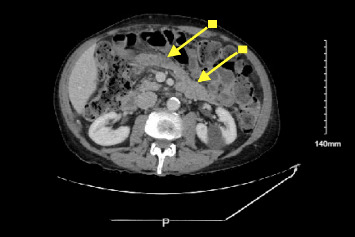
Axial CT scan of the abdomen and pelvis with contrast which was obtained after corticosteroid therapy for ICI-related pancreatitis was completed. The arrow is pointing at the pancreas, which now demonstrates normalization. No masses were perceived, and no lymphadenopathy was present. The peripancreatic fluid collections were described as decreasing.

Unfortunately, post-EUS, the patient developed symptoms of acute pancreatitis and was admitted 3 days later. He was treated symptomatically and started on prednisone 40 mg daily for type 3 AIP, with a subsequent slow taper. While taking the prednisone, his previously prescribed hydrocortisone and budesonide were held. His symptoms of microscopic colitis and pancreatitis resolved, and his amylase and lipase improved. PET CT obtained 1 year after his final cycle five of ICI therapy showed no evidence of F-fluorodeoxyglucose (FDG)-avid residual or recurrent malignancy.  Figure 5 is a contrast-enhanced CT image showing normalization of the pancreas. There were no masses or lymphadenopathy identified. Follow-up adrenal labs unfortunately showed persistent secondary adrenal insufficiency with a still undetectable morning cortisol level. At present, he continues on hydrocortisone therapy.

## 3. Discussion

Type 3 AIP is a diagnosis of exclusion, and in this patient, the common causes of pancreatitis had already been clinically ruled out. In most cases, pathology is not required to establish the diagnosis of type 3 AIP, as most can be diagnosed clinically. However, in this patient's case, the clinical presentation of masses in the pancreas raised concern for metastatic melanoma, primary pancreatic adenocarcinoma, or AIP. Therefore, FNB was very useful in distinguishing between malignancy and immune-induced injury, as it helped to guide therapy and to potentially avoid unnecessary surgical intervention.

Histologically, there was no evidence of a tumor. While lymphoplasmacytic infiltrates with ductal cuffing have been described in both type 1 and type 2 AIP, characteristic features suggestive of type 1 AIP (IgG4-positive plasma cells, storiform fibrosis with obliterative phlebitis) or type 2 AIP (neutrophil-rich acinar and ductal damage with intraductal luminal microabscesses or granulocyte epithelial lesions [GELs]) were absent [11]. Yet the pattern of CD4 and CD8+ T-lymphocytic infiltration into the acini and septal structures with acinar cell and duct epithelial cell dropout was strongly suggestive of immune-mediated damage. It in fact resembled the immunopathology seen in pancreatic allograft biopsies in the clinical setting of T-cell mediated rejection [12]. We also noted that the pattern of injury in the pancreas was similar to that often observed in the liver in cases consistent with ICI-related liver injury (CD4 and CD8 lymphocyte-mediated hepatocellular lobular and portal inflammation, ductitis, and venulitis) [13, 14]. Thus, CD3, CD4, and CD8 lymphocytic infiltration of pancreatic acini and interlobular septa with resultant acinar damage, ductitis, and venulitis likely represents an important histologic pattern of ICI-related pancreatitis (type 3 AIP), a condition that, to our knowledge, has only rarely been diagnosed on biopsy, resected specimens, or autopsy [15–30]. Upon review of the literature, summarized in Table 1, ICI-related pancreatic injury includes a spectrum of exocrine and endocrine-related inflammatory patterns. Exocrine injury presents with mixed CD4 and CD8 T-cell-mediated acinar and ductal damage of varying degrees [15–25] or rarely as a sclerosing/mass-forming IgG4-related pancreatitis, thus with histologic overlap with type 1 AIP [26]. Several studies describe neutrophil-predominant acinar inflammation, but GEL-like lesions have been described in only 2 cases; thus, there is little histologic overlap with type 2 AIP [15, 25]. Several studies describe acinar ductal metaplasia, a feature associated with marked acinar damage and atrophy, and some studies report granulomas composed of epithelioid histiocytes, neither of which were present in our case. In cases of ICI-related endocrine damage, all of which presented with diabetes, there is prominent CD8 T-cell infiltration of the pancreatic islet cells [27–29]. To our knowledge, the vascular damage/endotheliitis seen in our case has not been emphasized before. This may be an important finding to examine for, indicative of immune-mediated injury in small core biopsy samples.

Given the mode of action of PD-1/PD-L1 inhibitors, and in keeping with the ICI-related liver injury literature, a predominance of CD8 cells was expected. Instead, our case had relatively high numbers of CD4 cells in addition to a robust CD8 response. This is likely due to the fact that our patient received relatlimab, not ipilimumab. Relatlimab is a lymphocyte activation gene 3 (LAG3) inhibitor, known to enhance proliferation of effector CD4+ T-cell populations in vivo [30, 31]. Other authors have reported similar findings with ICI monotherapy. Chen et al. have speculated that varying numbers of CD4 and CD8 lymphocytes in the infiltrate might reflect temporal changes in the effector lymphocyte population depending on timing of the biopsy relative to the onset of ICI pancreatic injury, with greater CD4 numbers early on in the course of ICI injury and possibly higher CD8 counts later as the disease progresses [25]. One important caveat to be kept in mind, as emphasized by Hirota et al., is that core biopsies are prone to sampling artifact and certain features affecting larger ducts and vessels might only be discerned on resection or autopsy specimens, as the puncture route of biopsy avoids these structures [20]. Additional small and large samples, obtained under similar serendipitous circumstances, are likely to shed further light on the etiopathogenetic mechanisms in ICI-related pancreatitis.

ICIs have significant clinical benefit but are associated with irAEs, most commonly involving the skin, gastrointestinal system, liver, and endocrine systems [32]. Rarely, fulminant and even deadly toxicities can occur, which is why awareness, prompt recognition, and management are essential. irAEs are thought to occur due to an overall enhancement of the immune system, which is why temporary immunosuppression including glucocorticoid therapy can be an effective treatment. Gastrointestinal irAEs such as acute ICI colitis or hepatotoxicity are seen with greater frequency than type 3 AIP.

Classically, the incidence of asymptomatic lipase elevation (most often identified on routine bloodwork) in patients on ICI therapy has been reported to be roughly 3%-4% [3, 4, 10]. This incidence is thought to increase to up to 16% with longer duration of ICI therapy [33]. More recent studies have demonstrated different findings. In a retrospective study including 1069 patients who had received ICI therapy, Nagao et al. found a 1.8% rate of any pancreatic enzyme elevation, with only 0.5% having acute pancreatitis secondary to ICI therapy. Only one patient had severe acute pancreatitis resulting in death [8]. Townsend et al. assessed 6450 patients with cancer who had received ICI therapy and found 364 (5.6%) had at least one instance of an elevated serum lipase after ICI therapy initiation. Pancreatic injury was attributable to ICI use in only 105 (29%) of these patients—1.6% of the overall population who had received ICI therapy. Of the only 27 total patients with ICI-related acute pancreatitis, 4 (15%) presented asymptomatically with elevated lipase and pancreatic inflammation on imaging. In multivariable regression, the presence of other irAEs was positively associated with ICI-related acute pancreatitis. Of the 105 patients who developed ICI-related pancreatic injury, 3 (3%) developed exocrine insufficiency and 9 (9%) developed endocrine insufficiency, which were concentrated in those patients with ICI-related acute pancreatitis [34]. Thomas et al. retrospectively examined all adult patients who received ICI therapy and found 6763 had a normal lipase level, 76 had a lipase 2x ULN, and 248 had lipase 3x ULN. Of these 248 patients with lipase 3x ULN, 37% were symptomatic [35].

Lipase elevations can develop at any time throughout the treatment course, and there is not always a clear temporal relationship to treatment initiation [3]. Patients with symptomatic pancreatitis will have symptoms that are seen with other causes of pancreatitis, including fever, epigastric abdominal pain, nausea, vomiting, and diarrhea.

Thomas et al. additionally reviewed serial abdominal CT scans performed for follow-up of cancer in a random selection of patients who had either normal lipase (1x), lipase 2x ULN (2x), or lipase 3x ULN (3x), at multiple time points. They evaluated for changes in the pancreatic parenchymal density over time by calculating the pancreas-to-spleen attenuation ratio for each study. At the time of lipase elevation, a normal pancreas was seen in 92% of the 2x group and 67% of the 3x group. Independent of symptoms, 33% of the 3x group had at least one feature of pancreatitis on CT imaging, and 46% had visual volume loss of the pancreas. When volumetry of the pancreas was performed, the 1x group had no change in pancreatic volume seen pre-ICI vs. 1 year after ICI therapy. The 2x group demonstrated a decrease in median pancreatic volume from 3 months before lipase elevation to 1 year after ICI therapy. The 3x group demonstrated in increase in the median pancreatic volume at the time of pancreatitis, followed by decreased volume at 1 year post-ICI, which remained stable at 2 years post-ICI. Patients who received combined ICI therapy had greater volume loss at 1 year compared with those who received anti-PD-1 monotherapy. At 1-year post-ICI, 4% in the 1x group, 25% in the 2x group, and 56% in the 3x group developed at least 20% volume loss of the pancreas [35].

Long-term complications of ICI-related acute pancreatitis have been reported, including chronic pancreatitis, recurrent acute pancreatitis, diabetes, and exocrine pancreatic insufficiency [10]. The above cited study by Thomas et al. showed that in the lipase 3x ULN group, there was a significant increase in the pancreas/spleen attenuation ratio after ICI therapy compared with before. Along with the visualized pancreatic volume loss, this was considered suggestive of gland fibrosis. In the 1x and 2x groups, the increase in the pancreas/spleen attenuation ratio after vs. before ICI therapy was not statistically significant. Additionally, a new diagnosis of diabetes at the most recent follow-up was noted in 18.8% of the 3x group but 0% of the 2x group [35].

Our patient presented with vague symptoms and was found to have type 3 AIP. He had other irAEs related to his ICI therapy, including microscopic colitis and secondary adrenal insufficiency. Given his CT findings of infiltrative pancreatic masses, EUS-FNB was performed. Given his history, there was concern that these masses could represent metastatic melanoma or a new primary pancreatic adenocarcinoma. Pathology was instead consistent with a treatable cause, type 3 AIP, and he responded to corticosteroid therapy. Therefore, EUS with FNB changed management in this case and allowed for appropriate treatment of his immune-related adverse event.

## Figures and Tables

**Table 1:** A summary of ICI-related/type 3 AIP case reports/series with histologic findings.

Cases	ICI therapy/cancer type	Clinical features	Type of tissue sample	Pancreatic histology	IHC or ImFl findings	Pathogenetic type of pancreatic injury
Rawson et al. [15]	Pem/melanoma	Intestinal obstruction due to duodenal wall thickening next to pancreas, no serum IgG4 elevation	Whipple	Pancreatitis with increased neutrophils and lymphocytes; ducts with luminal neutrophils resembling GELs; acinar dropout and fibrosis	Not done	Type 3 AIP? Overlap with type 2 AIP

French et al. [16]	Nivo and Ipili/melanoma	Abdominal pain with elevated lipase and amylase	EUS-FNB	Acinar lymphocytic inflammation, with early fibrosis; no duct damage or venulitis	CD4+ and CD8+ T-cells; no IgG4 plasma cells	Type 3 AIP with T-cell-mediated acinar damage

Yoneda et al. [27] + Kawata et al. [28]	Nivo and Ipili/RCC	Metastatic RCC to pancreas followed by diabetic ketoacidosis	Pancreatectomy	Lymphocytes in pancreatic acini and focally within islet cells; beta cell depletion	CD3+ and CD8+ T-cells in islets	Type 3 AIP with endocrine pancreatic injury
	Pem/UrCA	New onset T1DM and elevated amylase and lipase	Autopsy			
	Nivo/HL	New onset T1DM	Autopsy			

Mazzucato et al. [29]	Pem/NSCLC	Diabetic hyperglycemia with ketoacidosis	Autopsy	Islet cell inflammation	Increased PDL-1 within pancreatic islets	Type 3 AIP with endocrine pancreatic injury

Janssens et al. [17]	Nivo/melanoma	Epigastric pain; elevated lipase; normal serum IgG4; enhancement of pancreas on CT-PET avid	EUS-FNB	Focal mild neutrophilic inflammation; fibrosis with no IgG4 plasma cells	Not done	Nonspecific AIP

Suda et al. [18]	Pem/lung CA	Liver injury and acute pancreatitis; on CT, pancreatic swelling and thickening of common bile duct	EUS-FNB	Acinar lymphocytic inflammation; no GELs	CD4+ & CD8+ T-cells; CD8+ > CD4+; no IgG4 plasma cells	Type 3 AIP with T-cell-mediated acinar damage

Tanaka et al. [19]	Nivo and Pazo/RCC	Abdominal pain with elevated lipase, amylase, and transaminases with pancreatic swelling on CT	Autopsy	Acinar necrosis with neutrophils and lymphocytes	Focal CD8+ and few CD4+ T-cells	Probable type 3 AIP T-cell-mediated acinar damage

Hirota et al. [20]	Pem/NSCLC	Abdominal pain; elevated amylase and lipase, diffuse enlargement of pancreas	EUS-FNB	Massive neutrophil infiltration with some lymphocytes and A-D-M; nonstoriform fibrosis and no obliterative phlebitis or GELs	Increased CD3+ T-cells; many CD8+ T-cells; no IgG4 plasma cells	Type 3 AIP with some histologic overlap with type 2 AIP

Song et al. [21]	Nivo/melanoma	Increased metabolic activity on PET in head and tail; chronic pancreatitis on EUS	EUS-FNB	Lymphocyte infiltration surrounding ducts and stromal fibrosis; no plasma cells; no GELs	Not done	Probable type 3 AIP with lymphocytic acinar damage

Ikemoto et al. [22]	Pem/NSCLC	Pancreatic head enlargement and hypoattenuation in body and tail; lipase and serum IgG4 normal	EUS-FNB	Lymphocytic infiltration of stroma with fibrosis	CD4+ and CD8+ T-cells; CD8+ > CD4+	Type 3 AIP; no overlap with type 1 or 2

Shi et al. [23]	Tori/adenoCA (clinical trial)	Epigastric discomfort; increased amylase and lipase; multiple lesions on MRI; normal IgG4	EUS-FNB	Architecture generally intact with focal pancreatic atrophy and neutrophilic and lymphocytic infiltrates without fibrosis; ducts preserved and no GELs	CD8 + and CD4+ T-cells; CD8+ cells >> CD4+ cells; few CD20+ B cells; no IgG4 plasma cells	Type 3 AIP with T-cell-mediated acinar damage

Champion et al. [26]	Pem/pleural mesothelioma	Pancreas mass on surveillance CT scan with distal duct dilatation	Whipple per prior FNAC with atypical cells	Storiform fibrosis; increased plasma cells; periductal stricture and obliterative phlebitis	Increased IgG4 plasma cells	Type 3 AIP with histologic overlap with type 1 AIP

Tanabe et al. [24]	Pem/endometrial carcinoma	Mass with normal lipase and normal serum IgG4	EUS-FNB	Lymphocytes amid extensive fibrosis; pancreatic acinar loss; A-D-M; prominent epithelioid granulomas; neutrophils sparse; no GELs	CD4+ and CD8+ T-cells; CD8+ = CD4+ without IgG4 plasma cells	Type 3 AIP with T-cell-mediated acinar damage

Chen et al. [25]	Pem/melanoma	Abdominal pain; elevated lipase and amylase with mass on imaging and new onset diabetes	Bx ×2	Atrophy; storiform fibrosis; increased lymphocytes and neutrophils; rare plasma cells; A-D-M+; no GELs or granulomas or phlebitis	CD4+ and CD8+ T-cells; CD4+ > CD8+	Type 3 AIP with impaired exocrine and endocrine function
	Pem/MSI-H colon CA	Asymptomatic mass on imaging with elevated lipase and amylase	Bx	Atrophy; increased lymphocytes and neutrophils; rare plasma cells and eosinophils; A-D-M; no GELs, granulomas, phlebitis, or fibrosis	CD4+ & CD8+ T-cells; CD8+ = CD4+	Type 3 AIP with T-cell-mediated acinar damage
	Nivo/pancreatic AdenoCA	Abdominal pain, diarrhea; amylase and lipase elevation	Autopsy	Lymphocytes predominant with some neutrophils, rare plasma cells; no GELs, granulomas or phlebitis; no A-D-M; no atrophy or storiform fibrosis	CD4+ and CD8+ T-cells; CD8+ > CD4+	Type 3 AIP
	Pem/UrCA	Abdominal pain; amylase and lipase elevation	Bx	Atrophy; mild lymphocytes and neutrophils; rare plasma cells and eosinophils; A-D-M; GEL-like +; no granulomas, phlebitis, or fibrosis	CD4+ and CD8+ T-cells; CD4+ > CD8+	Type 3 AIP? Overlap with type 2 AIP

Our case (2025); diagnosed in 1/2023	Nivo and relat/melanoma	Epigastric discomfort, elevated lipase: infiltrative mass lesions in the pancreas	EUS-FNB	Pancreatitis with predominant lymphocytes; conspicuous lymphocytic attack on acini, ducts, and vessels in interlobular septa with acinar dropout; no plasma cells, GELS, granulomas, A-D-M, storiform fibrosis, or obliterative phlebitis	CD4+ and CD8+ T-cells; CD4 >> CD8	Type 3 AIP (no overlap with type 1 or type 2)

*Note:* AdenoCA: adenocarcinoma; Bx: biopsy; CA: carcinoma; EUS-FNB: endoscopic ultrasound with fine needle biopsy (specifically referring to biopsy of the pancreas in this table); IHC: immunohistochemistry; ImFl: immunofluorescence; Ipili: ipilimumab; Nivo: nivolumab; Pazo: pazopanib; Pem: pembrolizumab; Relat: relatlimab; Tori: toripalimab; UrCA: urothelial CA.

Abbreviations: A-D-M = acinar-ductal-metaplasia, FNAC = fine needle aspiration cytology, GEL = granulocyte epithelial lesion, HL = Hodgkin lymphoma, MSI-H = microsatellite instability-high, NSCLC = nonsmall cell lung cancer, T1DM = type 1 diabetes mellitus.
